# Predicting Central Venous Pressure by Measuring Femoral Venous Diameter Using Ultrasonography

**DOI:** 10.7759/cureus.893

**Published:** 2016-11-23

**Authors:** Akram Malik, Aftab Akhtar, Shoab Saadat, Salman Mansoor

**Affiliations:** 1 Critical Care Medicine, Shifa International Hospital, Islamabad, Pakistan; 2 Department of Pulmonology and Critical Care, Shifa International Hospital, Islamabad, Pakistan; 3 Department of Nephrology, Shifa International Hospital, Islamabad, Pakistan; 4 Department of Neurology, Shifa International Hospital, Islamabad, Pakistan

**Keywords:** femoral venous diameter, central venous pressure, fvd, cvp, volume status, volume depleted patients, shock, septic shock, ivc

## Abstract

**Objectives:**

The objective of this exploratory study was to find out the correlation of femoral vein diameter (FVD) to central venous pressure (CVP) measurements and to derive a prediction equation to help ascertain the fluid volume status in a critical patient.

**Patients and methods:**

This was a single-centered prospective cohort study designed and conducted by the critical care department of Shifa International hospital in Islamabad, Pakistan. Patients were enrolled from the medical and surgical intensive care units. The inclusion criteria consisted of patients > 18 years of age, and an intrathoracic central venous catheterization (CVC) in place for producing CVP waveform through the transducer. Patients having contraindications to CVP placement and those unable to lie supine were excluded from the study. Critical Care fellows with sufficient training in performing venous ultrasonography measured the FVD. They were blinded to the CVP values of the same patients.

**Results:**

The study included 108 patients. Among these 70/108 (64.8%) were males. Mean age was 53.85 (SD=16.74). The CVP and femoral vein diameter were measured in all patients. Mean CVP was 9.89 cmH2O (SD=3.46) and mean femoral vein diameter was 0.92 cm (SD=0.27). Multiple regression was used to generate a prediction model. FVD, age and sex of the patient were used as predictor variables to predict CVP diameter. The model was statistically significant with a p-value of < 0.000 and an F-value of 104.806. R-squared value for this model came out to be 0.744, thus the model was able to explain about 74.4% of the variance in the values observed for CVP. When controlled for age and sex, FVD was found highly correlated with CVP diameter with a p-value of < 0.000. A regression equation was derived that can be used to generate predicted values of CVP in millimeters of mercury with an R-square of 0.745 if FVD in centimeters is provided; CVP (cmH2O) = -0.039 + 10.718* FVD.

**Conclusions:**

FVD was found highly correlated to CVP measurements and it suggests an alternate non-invasive method of ascertaining the volume status in the critically ill.

## Introduction

Fluid volume status in the management of the critically ill has been the cornerstone of any effective therapy. There are two available options to ascertain the volume status: invasive and noninvasive. There are some limitations for invasive volume status monitoring [1-3]. The current prevailing practices in intensive care units (ICUs) have been using central venous pressure (CVP) as a guide for fluid management; however, it is not the gold standard and sometimes is misleading [4]. Pulmonary artery catheterization has been the lesser employed invasive modality due to risks and the technical expertise it requires to monitor the fluid status, but studies have not proved any better outcome with it [5]. The common complications associated with pulmonary artery catheterization (PAC) are infection, thrombosis and vascular injury [2].

Among the noninvasive techniques, measuring inferior vena cava diameter (IVC) by ultrasonography has been proved reliable to convey information about the vascular volume [3, 6-7]. IVC diameter and its collapsibility index (IVC-CI) show good correlation with volume status, and clinical interventions can be made based on IVC sonography [8-10]. Sometimes IVC-CI assessment by ultrasound is difficult especially in circumstances where there is abdominal distension, abdominal wounds, external compression by masses, increased intra-abdominal pressure, and morbid obesity [1, 3-9].

Another way of assessing CVP monitoring noninvasively is ultrasonography of the femoral vein. This vein is superficial with no significant anatomical obstruction and is easily imaged. Previous studies have shown a strong correlation of common iliac vein pressure with CVP [11-13].

## Materials and methods

This was a single-centered prospective cohort study designed and conducted by the critical care department of Shifa International hospital in Islamabad, Pakistan. Patients were enrolled from the medical and surgical intensive care units. The study was approved by the ethics and review board of Shifa International hospital under the approval number of 606-054-2016. Written, informed consent was obtained from patients and their caregivers when the patients were unable to consent for themselves. The inclusion criteria consisted of patients > 18 years of age, and an intrathoracic central venous catheterization (CVC) in place for producing CVP waveform through the transducer. Patients having contraindications to CVP placement and those unable to lie supine were excluded from the study. Patients with a history of femoral catheterization were kept in the study as the femoral vein on the other side could also be used for FVD measurement. 

**Figure 1:**
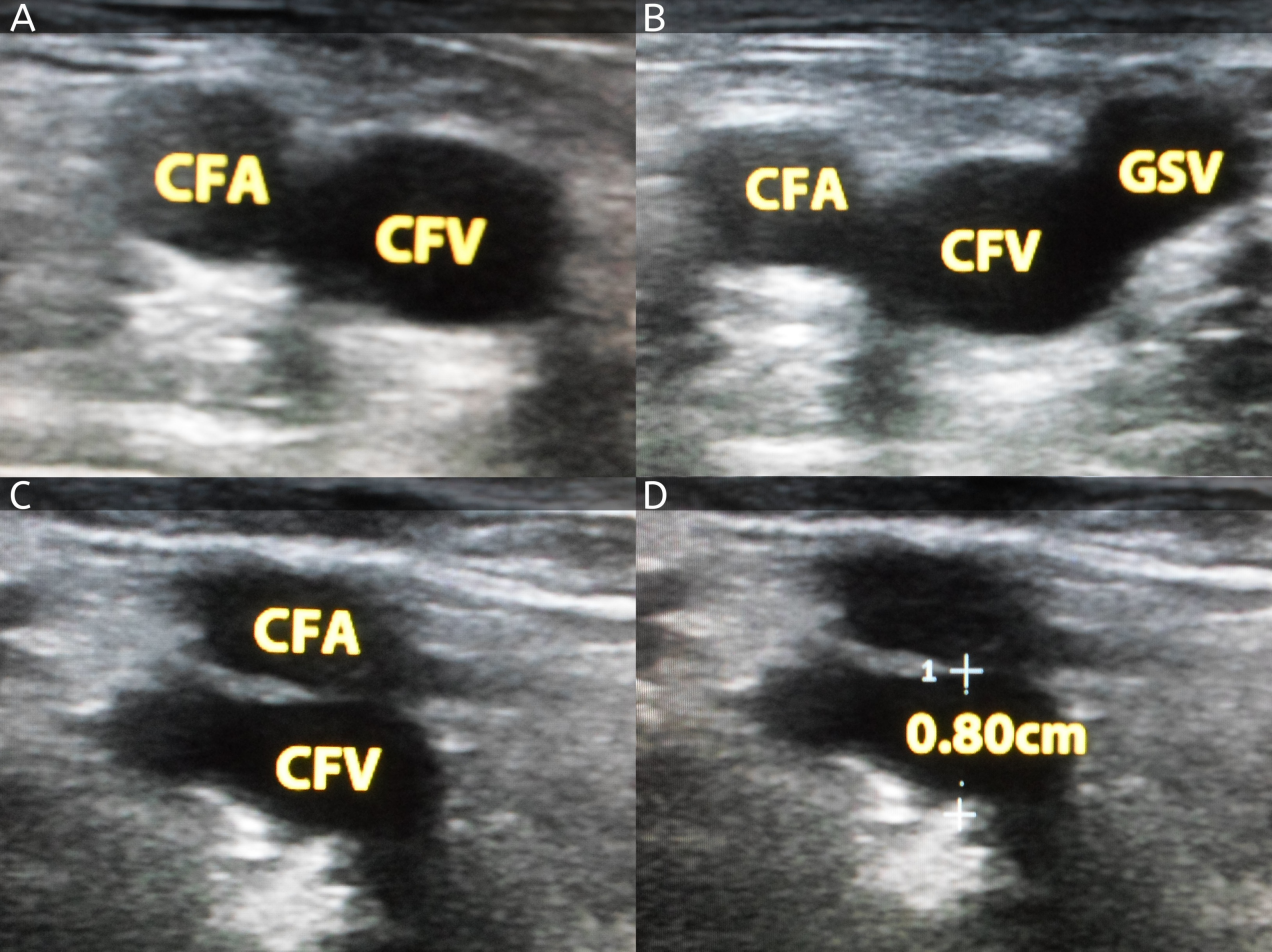
Ultrasonographic images for various measurements A. Ultrasound image of common femoral artery (CFA) and common femoral vein (CFV) taken at inguinal ligament transversely. B. The great saphenous vein (GSV) joining the common femoral vein. C. Image taken at distal site of CFV where the GSV disappears (the point where FVD was measured). D. FVD was measured anteroposteriorly at the center.

Critical care fellows were formally given an orientation for the procedure protocols to minimize operator-related bias. Two of the fellows who had completed the required competencies in the procedure were allowed to perform the ultrasound. The sonographer was blinded for the CVP readings before performing the femoral ultrasound. Patients were kept in a supine position during the full course of the ultrasound. Mindray diagnostic ultrasound system model Z6 ultrasound machine (Mindray, South Carolina, USA) was used for obtaining images (Figure 1).

By using the inguinal ligament crease, the right common femoral vein was identified [14]. After applying ultrasound gel, the great saphenous vein take off was traced by scanning caudally at the anterior medial aspect of the common femoral vein. When the great saphenous vein was no longer seen on ultrasonography caudally, the femoral vein diameter (FVD) was measured. It was measured by using leading edge technique at the anteroposterior dimension of the common femoral vein. To confirm the presence of the vein, compression technique was used and deep venous thrombosis (DVT) was also excluded [15]. To check CVP it is mandatory to zero the pressure transducer and keep the patient supine; so it was done accordingly. There was no effect of the respiratory cycle on the imaging of femoral vein diameter. CVP was taken from ECG lead 11 and paper strip reading with transducer was done at mid-axillary position. CVP was recorded at end-expiration. A previous study took a CVP cut off of less than 10 mmHg for predicting CVP non-invasively [16]. We used CVP diameter as a continuous variable for the multiple regression models. 

Data analysis was done on SPSS 24 (IBM Corp., NY, USA ). Results were reported as means ± standard deviation (SD). A p value of < 0.05 was taken as statistically significant. Multiple regression was used to generate a prediction model for estimating CVP if FVD values were provided.

## Results

The study included 108 patients. Out of the 108, 70 (64.8%) were males. The mean age was 53.85 (SD=16.74). The CVP and femoral vein diameter were measured in all patients. The mean CVP was 9.89 cm H2O (SD=3.46) and the mean femoral vein diameter was 0.92 cm (SD=0.27). The minimum mean CVP value was 3 cmH2O and the maximum mean CVP was 23 cmH2O. While the minimum mean femoral vein diameter was 0.21 cm and the maximum mean FVD was 1.73 cm.

For inferential statistics, a multiple regression model was generated (Table 1).

**Table 1:** Coefficients table

	Unstandardized Coefficient B	t	Significance level	95.0% Confidence Interval for B	
				Lower Bound	Upper Bound
(Constant)	-0.756	-0.728	0.468	-2.815	1.302
Femoral Venous Diameter	10.813	17.689	0	9.601	12.025
Age	0.002	0.01	0.992	-0.393	0.397
Gender	0.46	1.282	0.203	-0.252	1.173
Dependent Variable: Central Venous Pressure					
R Square value: 0.751					

**Figure 2:**
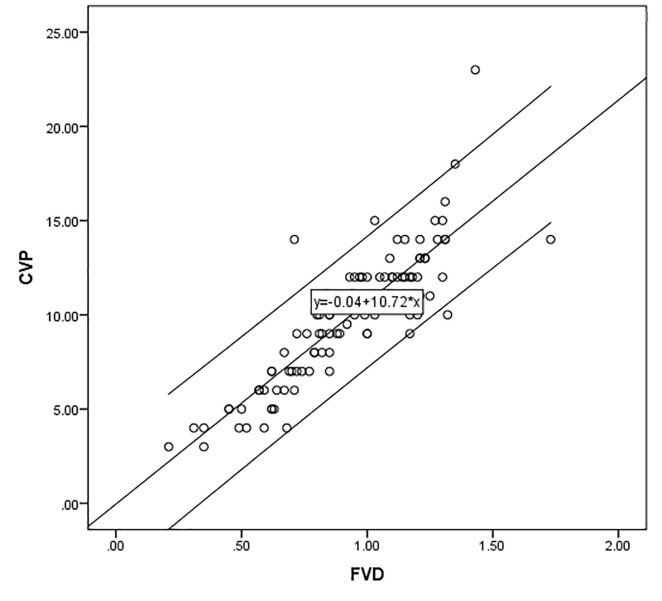
Scatter plot with line of best fit for femoral venous diameter and central venous pressure FVD: Femoral venous diameter CVP: Central venous pressure

All the assumptions for this model were met, including the normal distribution of data and checking for outliers using Cook’s distances. Multicollinearity was ruled out by testing for correlations between the predictor variables themselves. FVD, age and sex of the patient were used as predictor variables to predict CVP. The model was statistically significant for analysis of variance (ANOVA), with a p-value of < 0.000 (Table 2) and an F-value of 104.806. Adjusted R-square value for this model came out to be 0.744, thus the model was able to explain about 74.4% of the variance in the values observed for CVP (dependent variable), which also meant around 50% of the standard deviation explained in comparison to a constant model with mean values for all the predictor variables. When controlled for age and sex, FVD was found highly correlated with CVP with a p-value of < 0.000. Using a more simplified version of the above mentioned model that contained FVD as the only predictor, a regression equation was derived that can be used to generate predicted values of CVP in centimeters with an adjusted R-square of 0.745 if FVD in centimeters is provided; CVP (cmH2O) = -0.039 + 10.718*FVD (cms). The scatter plot with the line of best fit is shown below in Figure [2].

A further subgroup analysis was carried out to find the regression patterns among patients with different ages and sexes. Several models were generated for each sex and different age groups. It was found that females had a stronger correlation between FVD and CVP with an R-square of 0.861 and a p-value of < 0.000. It was also found that among the patients of ages between 21-40 years, an even stronger model was generated with an R-square of 0.872 and p-value of < 0.000.

**Table 2:** ANOVA test for the model

Model		df	Mean Square	F	Sig.
1	Regression	3	321.625	104.807	<0.000a
	Residual	104	3.069		
	Total	107			
Dependent Variable: CVP					
a. Predictors: (Constant), FVD, Age, Gender					

 

## Discussion

A multivariate regression model was able to predict with a high R-square (0.751) the CVP in centimeters if FVD values are provided. This holds true for both the sexes and for all the age groups as well. A subgroup analysis was able to point out that an even stronger association was found between the two variables in females and in patients between 21 to 40 years of age. This may be due to the fact that both younger and female patients tend to have lesser subcutaneous fat deposition thus allowing for better visualization of the femoral vein and reduction in measurement bias. CVP is placed for diagnostic measurement, monitoring and knowing the fluid status of patients in ICU and interventions accordingly [17]. Like all procedures, this procedure is also associated with some complications. Bedside sonography seems to be an emerging replacement for invasive CVP procedure for knowing intravascular volume status of patients in ICU. IVC and internal jugular vein examination by ultrasound have been used in some studies for estimating CVP [18-23]. One such study has shown that maximal IVC diameter is a better way of assessing CVP than IVC collapsibility index or internal jugular vein aspect ratio in spontaneously breathing patients [16]. Sometimes assessing IVC by ultrasound is difficult because of abdominal distension, abdominal wound or external compression by masses. Also, there is the possibility of errors in measuring IVC diameter or collapsibility index because of the respirophasic movement of IVC [24].

In this study, measurement of the FVD has been used for estimating CVP. Previously, some studies have been performed which show different degrees of correlation. One such study by Roy J, et al. conducted between November 2012 and February 2013 showed a moderate degree of correlation between the two parameters. This study included 108 patients; 64.8% were males and 35.2% were females. The mean CVP was 9.89 cm H2O and the mean FVD was 0.92 cm. The results showed a strong correlation between CVP and FVD (p < 0.000).

There are some limitations in this study. Firstly, this was a single-center study and included mostly Pakistani patients, which may lead to a compromise in its external validity or generalizability. Secondly, a small sample size may not allow the regression equation to be highly applicable to a wide variety of population and there is always a chance of overfitting in such conditions. Thirdly, this study included a small proportion of both surgical and medical ICU patients and patients on and off ventilator. A subgroup analysis in these small subsets of groups will lead to a compromised power. More studies with greater sample sizes may help resolve this limitation.

## Conclusions

FVD was found highly correlated to CVP measurements and it suggests an alternate noninvasive method of ascertaining the volume status in the critically ill.

## References

[1] Stawicki SP, Braslow BM, Panebianco NL, Kirkpatrick JN, Gracias VH, Hayden GE, Dean AJ (2009). Intensivist use of hand-carried ultrasonography to measure IVC collapsibility in estimating intravascular volume status: correlations with CVP. J Am Coll Surg.

[2] Evans DC, Doraiswamy VA, Prosciak MP, Silviera M, Seamon MJ, Rodriguez Funes V, Cipolla J, Wang CF, Kavuturu S, Torigian DA, Cook CH, Lindsey DE, Steinberg SM, Stawicki SP (2009). Complications associated with pulmonary artery catheters: a comprehensive clinical review. Scand J Surg.

[3] Carr BG, Dean AJ, Everett WW, Ku BS, Mark DG, Okusanya O, Horan AD, Gracias VH (2007). Intensivist bedside ultrasound (INBU) for volume assessment in the intensive care unit: a pilot study. J Trauma Acute Care Surg.

[4] Marik PE, Baram M, Vahid B (2008). Does central venous pressure predict fluid responsiveness? A systematic review of the literature and the tale of seven mares. Chest.

[5] Shah MR, Hasselblad V, Stevenson LW, Binanay C, O'Connor CM, Sopko G, Califf RM (2005). Impact of the pulmonary artery catheter in critically ill patients: meta-analysis of randomized clinical trials. Jama.

[6] Moreno FL, Hagan AD, Holmen JR, Pryor TA, Strickland RD, Castle CH (1984). Evaluation of size and dynamics of the inferior vena cava as an index of right-sided cardiac function. Am J Cardiol.

[7] Kent A, Patil P, Davila V, Bailey JK, Jones C, Evans DC, Boulger CT, Adkins E, Balakrishnan JM, Valiyaveedan S, Galwankar SC, Bahner DP, Stawicki SP (2015). Sonographic evaluation of intravascular volume status: can internal jugular or femoral vein collapsibility be used in the absence of IVC visualization?. Ann Thorac Med.

[8] Ferrada P, Anand RJ, Whelan J, Aboutanos MA, Duane T, Malhotra A, Ivatury R (2012). Qualitative assessment of the inferior vena cava: useful tool for the evaluation of fluid status in critically ill patients. Am Surg.

[9] Ando Y, Yanagiba S, Asano Y (1995). The inferior vena cava diameter as a marker of dry weight in chronic hemodialyzed patients. Artif Organs.

[10] Seif D, Perera P, Mailhot T, Riley D, Mandavia D (2012). Bedside ultrasound in resuscitation and the rapid ultrasound in shock protocol. Crit Care Res Pract.

[11] Ho KM, Joynt GM, Tan P (1998). A comparison of central venous pressure and common iliac venous pressure in critically ill mechanically ventilated patients. Crit Care Med.

[12] Nahum E, Dagan O, Sulkes J, Schoenfeld T (1996). A comparison between continuous central venous pressure measurement from right atrium and abdominal vena cava or common iliac vein. Intensive Care Med.

[13] Cho RJ, Williams DR, Leatherman JW (2016). Measurement of femoral vein diameter by ultrasound to estimate central venous pressure. ATS Journals.

[14] Coleridge-Smith P, Labropoulos N, Partsch H, Myers K, Nicolaides A, Cavezzi A (2006). Duplex ultrasound investigation of the veins in chronic venous disease of the lower limbs-UIP consensus document. Part I. Eur J Vasc Endovasc Surg.

[15] Kory PD, Pellecchia CM, Shiloh AL, Mayo PH, DiBello C, Koenig S (2011). Accuracy of ultrasonography performed by critical care physicians for the diagnosis of DVT. Chest.

[16] Prekker ME, Scott NL, Hart D, Sprenkle MD, Leatherman JW (2013). Point-of-care ultrasound to estimate central venous pressure: a comparison of three techniques. Crit Care Med.

[17] Scales K (2010). Central venous pressure monitoring in clinical practice. Nurs Stand.

[18] Kircher BJ, Himelman RB, Schiller NB (1990). Noninvasive estimation of right atrial pressure from the inspiratory collapse of the inferior vena cava. Am J Cardiol.

[19] Brennan JM, Blair JE, Goonewardena S, Ronan A, Shah D, Vasaiwala S, Kirkpatrick JN, Spencer KT (2007). Reappraisal of the use of inferior vena cava for estimating right atrial pressure. J Am Soc Echocardiogr.

[20] Schefold JC, Storm C, Bercker S, Pschowski R, Oppert M, Kruger A, Hasper D (2010). Inferior vena cava diameter correlates with invasive hemodynamic measures in mechanically ventilated intensive care unit patients with sepsis. J Emerg Med.

[21] Nagdev AD, Merchant RC, Tirado-Gonzalez A, Sisson CA, Murphy MC (2010). Emergency department bedside ultrasonographic measurement of the caval index for noninvasive determination of low central venous pressure. Ann Emerg Med.

[22] Donahue SP, Wood JP, Patel BM, Quinn JV (2009). Correlation of sonographic measurements of the internal jugular vein with central venous pressure. Am J Emerg Med.

[23] Simonson JS, Schiller NB (1988). Sonospirometry: a new method for noninvasive estimation of mean right atrial pressure based on two-dimensional echographic measurements of the inferior vena cava during measured inspiration. J Am Coll Cardiol.

[24] Blehar DJ, Resop D, Chin B, Dayno M, Gaspari R (2012). Inferior vena cava displacement during respirophasic ultrasound imaging. Crit Ultrasound J.

